# An Insight into the Sialome of the Lone Star Tick, *Amblyomma americanum*, with a Glimpse on Its Time Dependent Gene Expression

**DOI:** 10.1371/journal.pone.0131292

**Published:** 2015-07-01

**Authors:** Shahid Karim, José M. C. Ribeiro

**Affiliations:** 1 Department of Biological Sciences, The University of Southern Mississippi, Hattiesburg, Mississippi, United States of America; 2 Vector Biology Section, Laboratory of Malaria and Vector Research, National Institute of Allergy and Infectious Diseases, National Institutes of Health, Rockville, Maryland, United States of America; Metabiota, UNITED STATES

## Abstract

Hard ticks feed for several days or weeks on their hosts. Blood feeding is assisted by tick saliva, which is injected in the host skin regularly, alternating with blood ingestion. Tick saliva contains hundreds or thousands of different peptides and other bioactive compounds that assist feeding by inhibiting their hosts’ blood clotting, platelet aggregation, vasoconstriction, as well as pain and itching. Immunomodulatory and antimicrobial peptides are also found in tick saliva. Molecular characterization of tick salivary compounds, or its sialome (from the Greek sialos = saliva), helps identification of possible antigens that might confer anti-tick immunity, as well as identifying novel pharmacologically active compounds. *Amblyomma americanum* is a major nuisance tick in Eastern and Southern US, being a vector of *Theileria* and *Ehrlichia* bacteria to animals and humans. Presently we report an RNAseq study concerning the salivary glands of adult female *A*. *americanum* ticks, which involved sequencing of four libraries collected at different times of feeding. A total of 5,792 coding sequences were deduced from the transcriptome assembly, 3,139 of which were publicly deposited, expanding from the previously available 146 salivary sequences found in GenBank. A remarkable time-dependent transcript expression was found, mostly related to secretory products, supporting the idea that ticks may have several “sialomes” that are expressed at different times during feeding. The molecular nature of this sialome switching remains unknown. The hyperlinked spreadsheet containing the deduced coding sequences can be found at http://exon.niaid.nih.gov/transcriptome/Amb_americanum/Ambame-web.xlsx.

## Introduction

Hard ticks feed for several days or weeks on their vertebrate hosts, acquiring a very large meal relative to their size, which may be over 100 fold their pre-engorgement weight. Tick feeding is usually divided into three phases associated with attachment, slow feeding and fast feeding phases [1]. Tick attachment involves the penetration of the skin by the chelicera and secretion of salivary cement that helps to fix the tick at the feeding site. Tick saliva also assists feeding through its complex mixture of compounds that disarms their hosts’ hemostasis, which includes platelet aggregation, vasoconstriction and blood clotting, as well as their innate and acquired immunity arms of inflammation in the form of salivary anticomplement and lymphocyte inhibitors, among other activities; antimicrobial peptides have also been found in tick saliva, perhaps controlling microbial infection at the tick bite and in the ingested meal [2–4]. Tick saliva composition as revealed by sialotranscriptomes (from the Greek, sialo = saliva) indicates the existence of over 1,000 putative secreted peptides grouped into dozens of protein families [3]. It appears that during the prolonged feeding period of ticks, which may last for weeks, different members of related proteins are expressed at distinct times thus possibly avoiding their hosts’ immune response [5].

The tick *Amblyomma americanum* is a three host tick, widespread in the Eastern and Southeastern United States, being a major nuisance and confirmed vector of tularemia and ehrlichiosis to humans and animals, and *Theileria cervi* to white deer [6, 7]. *Amblyomma americanum* bites additionally are a possible cause of IgE antibody responses to α-gal in the southeastern United States [8]. The current known distribution of delayed anaphylactic reactions to red meat is similar to known *A*. *americanum* distribution. Previously, a sialotranscriptome of this tick has been reported consisting of 3,869 expressed sequence tags (EST’s) from which 142 coding sequences (CDS) have been deposited to GenBank [9]. We presently report an extension of this sialome by assembling over 344 million Illumina sequences totaling over 34 billion nucleotides deriving from four salivary gland cDNA libraries made from *A*. *americanum* adult female ticks that were unfed, or fed for 12–48 h, 72–144 h and 7–11 days, allowing identification of the changing profile of transcription in salivary glands as feeding progresses. We added 3,139 deduced protein sequences to the Transcriptome Shotgun Annotation (TSA) portal of the National Center for Biotechnology Information (NCBI), providing for a discovery platform for proteomic studies that will serve to mine proteins of antigenic or pharmacological interest. We additionally describe the remarkable change in transcription levels of hundreds of transcripts according to time of feeding, most of a secreted nature, and discuss the possible mechanisms of sialome switching in feeding ticks.

## Material and Methods

### Ethics statement

All animal experiments were performed in strict accordance with the recommendations in the Guide for the Care and Use of Laboratory Animals of the National Institutes of Health. The protocol of tick blood feeding on the sheep was approved by the Institutional Animal Care and Use Committee of the University of Southern Mississippi (protocol # 10042001). All efforts were made to minimize animal suffering.

### Ticks and other animals

The lone star ticks (*A*. *americanum*) were maintained at the University of Southern Mississippi according to established methods [10]. Unfed adult ticks were obtained from Oklahoma State University’s tick rearing facility (Stillwater, OK) for this study. Unfed ticks were maintained at room temperature and 90% relative humidity under 14/10 hour light/dark photoperiod before infestation on sheep. Ticks were fed on sheep and were either allowed to feed to repletion or removed between 12–264 hours, depending on the experimental protocol. Adult ticks were blood-fed specifically for this study and animal studies were performed in accordance with protocols approved by the Institutional Animal Care and Use Committee (IACUC) at the University of Southern Mississippi.

### Tick salivary glands (SG) preparation

The unfed and blood-fed (12, 18, 24, 36, 48, 72, 120, 144, 168, 192, 216, 264 hrs post attachment) female adult ticks were dissected within four hours of after removal from the sheep. Tick salivary glands were dissected in ice-cold M-199 buffer [11, 12]. After removal, salivary glands were washed gently in the same ice-cold buffer. The dissected SGs were stored immediately after dissection in RNAlater (Invitrogen, Carlsbad, CA, USA) prior to extracting mRNA.

### RNA Preparation

Total RNA was isolated from salivary glands dissected from unfed and partially blood-fed adult female ticks using illustra RNAspin Mini RNA isolation kit (GE Healthcare, NJ, USA) following the manufacturer’s protocol. The quality of the RNA samples was confirmed by lab-on-chip analysis using the 2100 Bioanalyzer (Agilent Technologies, Inc. Santa Clara, CA, USA). The total RNA quantity was determined by a Nanodrop, and the total RNA samples (A260/280> 1.8–2) were pooled into 4 groups (Group I, UF: Unfed SGs only; Group II, 12–48 h: 12, 18, 24, 36, 48 hours post attachment; Group III, 72–144 h: 72, 120, 144 hours post attachment and Group IV, 7–11 days: 168, 192, 216, 264 hours post attachmen) for further cDNA synthesis.

### Illumina Sequencing

Total RNA samples were submitted to Otogenetics Corporation (Norcross, GA USA) for RNA-seq assays. Total RNA was treated with the Ambion GLOBINclear-Human Kit to deplete globin mRNA that may have resulted from the blood meal; the Clontech SmartPCR cDNA kit (Clontech Laboratories, Inc, Mountain View, CA USA, Cat#634926) was used to generate 1–2 μg of cDNA from 100 ng of total RNA, following the manual instructions that included 6 min at 68°C followed by 10 min at 70°C during the 14 cycles of the PCR reaction. Restriction digestion was used to remove adaptor sequences and the resulting cDNA was fragmented using Covaris (Covaris, Inc., Woburn, MA USA), profiled using Agilent Bioanalyzer, and subjected to Illumina library preparation using NEBNext reagents (New England Biolabs, Ipswich, MA USA). Agilent Bioanalzyer 2100 was used to assess the quality, quantity, and size distribution of the Illumina libraries. The libraries were then submitted for Illumina HiSeq2000 sequencing according to the standard operation. Paired-end 90 or 100 nucleotide reads were generated and checked for data quality using FASTQC (Babraham Institute, Cambridge, UK). The number of paired ended reads of 100 nt in length for each of the four libraries was: Group I, 106,249,828; Group II 50,190,144; Group III, 99,582,182 and Group IV, 88,887,224 reads. Sequence reads were deposited to the NCBI under Bioproject PRJNA218793, Biosample SAMN02352759 and read files SRR1740607 (unfed), SRR1740608 (12–48h), SRR1740609 (72–144 h) and SRR1740611 (7–11 days).

### Bioinformatics Analysis

Assembly of all reads was done using the assemblers Abyss and Soapdenovo-Trans with every other kmer (-k program switch) parameter from 17 to 85 [13–17]. Resulting contigs were re-assembled by a pipeline of blastn and cap3 assembler [18] as described earlier [19]. Coding sequences were extracted based on blastx [20] results deriving from several database matches, including a subset of the non-redundant protein database of the National Center for Biotechnology Information (NCBI) containing tick and other invertebrate sequences, as well as the Swissprot and Gene Ontology (GO) databases. The longest open reading frame was also extracted if it had a signal peptide indicative of secretion as evaluated by version 3.0 of the SignalP program [21]. Reads from the four libraries were mapped back into the CDS by blastn with a word size of 25 and allowing one gap. Reads were mapped up to a maximum of five different CDS if the blast scores were the same for all matches. Read number differences for each CDS between libraries were compared by a X^2^ test. For heat map display [22] of the CDS temporal expression, the number of reads for each treatment was normalized by multiplying it to the grand total number of reads deriving from all libraries and dividing the product by the total number of reads of the particular library, zeroes were replaced by one, then each row of data was average normalized, and then log(10) transformed. Heatmaps were produced with the programs gplots and heatmap.2 using R [23]. Differential gene expression clustering was done with the program Expander version 6.5 [24], using as input read fragments per thousand nucleotides per million (FPKM) data and the click1 algorithm. More details of the input are available in the results section.

All coding sequences and their reads are available for browsing in the supporting S1 File which also contains hyperlinks to several databases, as explained previously [19, 25]. FPKM were calculated for each library [26]. Deduced coding sequences and their translations were deposited at DDBJ/EMBL/GenBank under the accession GBZX00000000. The version described in this paper is the first version, GBZX01000000.

## Results and Discussion

Assembly of the 344,909,378 reads from 4 libraries allowed extraction of 5,792 CDS that mapped 143,158,375 reads. Following blast and rpsblast comparisons to several databases, these CDS were classified into 27 categories revealing 49% of the total reads mapping to CDS associated with putative secreted proteins followed by 18% of reads mapping to CDS associated with the protein synthesis machinery, a common finding in previous tick sialotranscriptomes (Table 1). Transposable elements and bacterial/algal sequences were also identified. The secreted CDS group was further classified into 8 major subdivisions as indicated in Table 2. Noticeable are the abundance of members of the Lipocalin group (200 CDS and 27% of the reads of the secreted class) as well as of the Kunitz group (over 100 CDS and > 8% of the reads). The subclass of Glycine rich proteins, which includes cement proteins, contains 46 CDS mapping 16% of the reads. Several previously orphan protein families were deorphanized and novel protein families were discovered. Consult previous publications for further information on the different protein families described in Table 2 [3, 19]. The hyperlinked spreadsheet containing all CDS and their classifications can be found in the supporting S1 File. Three thousand and forty one CDS and their translations were deposited in the TSA portal of the NCBI.

**Table 1:** Functional classification and associated mapped reads deriving from the sialotranscriptome of *Amblyomma americanum*.

Class	Number of CDS	Percent of CDS	Number of Reads	Percent of Reads
Secreted	2,153	37.172	69,544,089	48.578
Protein synthesis machinery	251	4.334	25,125,390	17.551
Unknown, conserved	494	8.529	16,195,994	11.313
Metabolism, energy	129	2.227	7,390,428	5.162
Protein export machinery	165	2.849	4,747,170	3.316
Unknown	636	10.981	3,004,072	2.098
Signal transduction	315	5.439	2,889,793	2.019
Protein modification machinery	164	2.831	2,734,514	1.910
Transporters/storage	176	3.039	2,447,877	1.710
Transcription machinery	249	4.299	1,408,049	0.984
Cytoskeletal	92	1.588	1,331,574	0.930
Proteasome machinery	121	2.089	1,315,949	0.919
Metabolism, carbohydrate	89	1.537	1,018,648	0.712
Oxidant metabolism/detoxification	63	1.088	741,259	0.518
Nuclear regulation	115	1.985	655,105	0.458
Storage	14	0.242	555,726	0.388
Metabolism, lipid	115	1.985	534,638	0.373
Transcription factor	69	1.191	405,461	0.283
Metabolism, nucleotide	66	1.140	241,402	0.169
Metabolism, amino acid	32	0.552	198,182	0.138
Immunity	27	0.466	150,807	0.105
Extracellular matrix/cell adhesion	39	0.673	140,583	0.098
Metabolism, intermediate	27	0.466	133,177	0.093
Nuclear export	17	0.294	52,429	0.037
Transposable element	97	1.675	87,648	0.061
Bacterial	59	1.019	86,794	0.061
Algal	18	0.311	21,617	0.015
**Total**	5,792	100.000	143,158,375	100.000

**Table 2:** Functional classification and associated mapped reads deriving from the CDS associated with putative secreted proteins found in the sialotranscriptome of *Amblyomma americanum*.

Class	No. of CDS	No. of Reads	Percent of Reads
Enzymes			
Salivary 5'-nucleotidase	3	5,118	0.007
Endonuclease	2	14,601	0.021
Carboxypeptidase	10	179,505	0.258
Metalloprotease reprolysin family	29	310,556	0.447
Metalloprotease M13 family	18	898,131	1.291
Phospholipase	5	18,696	0.027
Protease inhibitor domains			
Serpins	10	23,169	0.033
Kunitz superfamily	85	5,330,593	7.665
Kunitz 45/50 family	14	462,788	0.665
Additional Kuniz peptides	46	815,657	1.173
Carboxyopeptidase inhibitor	1	1,144	0.002
Cystatin	13	144,850	0.208
Thyropin	3	18,331	0.026
TIL domain polypeptides	29	1,354,404	1.948
TIL II family	14	2,027,658	2.916
Lipocalins	200	18,815,797	27.056
Immunity related peptides			
Leucine rich Toll like proteins	2	11,526	0.017
ML domain containing protein	5	10,293	0.015
Galectin	3	4,201	0.006
Lysozyme	2	132,879	0.191
Microplusin	11	124,504	0.179
Possible defensin	8	25,590	0.037
5.3 kDa peptide	23	161,938	0.233
Ubiquitous domains, function unknown			
Antigen 5 SCP family	3	13,647	0.020
Tick specific families—known function			
Glycine rich proteins, possible cement	46	11,177,996	16.073
Glycine rich small peptide	14	514,224	0.739
Cement-like protein	4	1,247,971	1.795
Peritrophin/Cuticle	4	18,458	0.027
Mucins	28	327,369	0.471
Evasin	43	625,939	0.900
Tick specific families—unknown function			
Insulin growth factor binding family	1	1,808	0.003
23 kDa family	2	4,929	0.007
28 kda metastriate family	6	22,161	0.032
One of each family	12	28,339	0.041
Ixostatin family	8	1,236,225	1.778
10 kDa acidid metastriate family	8	212,768	0.306
18.3 kDa family	9	548,942	0.789
Ixodegrin	14	66,559	0.096
8 kDa Amblyomma peptide	25	2,598,552	3.737
Basic tail	5	816,944	1.175
8.9 kDa family	27	1,819,462	2.616
8.9 kDa family 45–15	11	35,028	0.050
8.9 kDa family 45–22	9	2,279,320	3.278
Other members of the 8.9 kDa superfamily	98	2,407,249	3.461
8.5 kDa family	13	81,919	0.118
Small basic tail family	10	93,134	0.134
Tick specific family I	6	234,980	0.338
Other tick-specific salivary proteins	80	1,574,657	2.264
Other tick-specific salivary proteins, mostly fragments or novel	1,030	8,181,421	11.764
Novel or deorphanized families			
Novel family 40–17	17	843,358	1.213
Pulchellus peptide family I	7	101,687	0.146
Pulchellus peptide family II	7	21,169	0.030
Pulchellus peptide family III	6	57,251	0.082
Ambvar protein family	20	1,326,506	1.907
Amlyomma specific family I	9	33,883	0.049
Ambam specific family II	12	3,148	0.005
Ambam specific family III	3	1,622	0.002
Ambam specific family IV	4	573	0.001
Ambam specific family V	4	4,734	0.007
Ambam specific family VI	6	4,899	0.007
Ambam specific family VII	6	83,329	0.120
**Total**	2,153	69,544,089	100.000

### Differential gene expression following feeding according to RNAseq data

**Fig 1:**
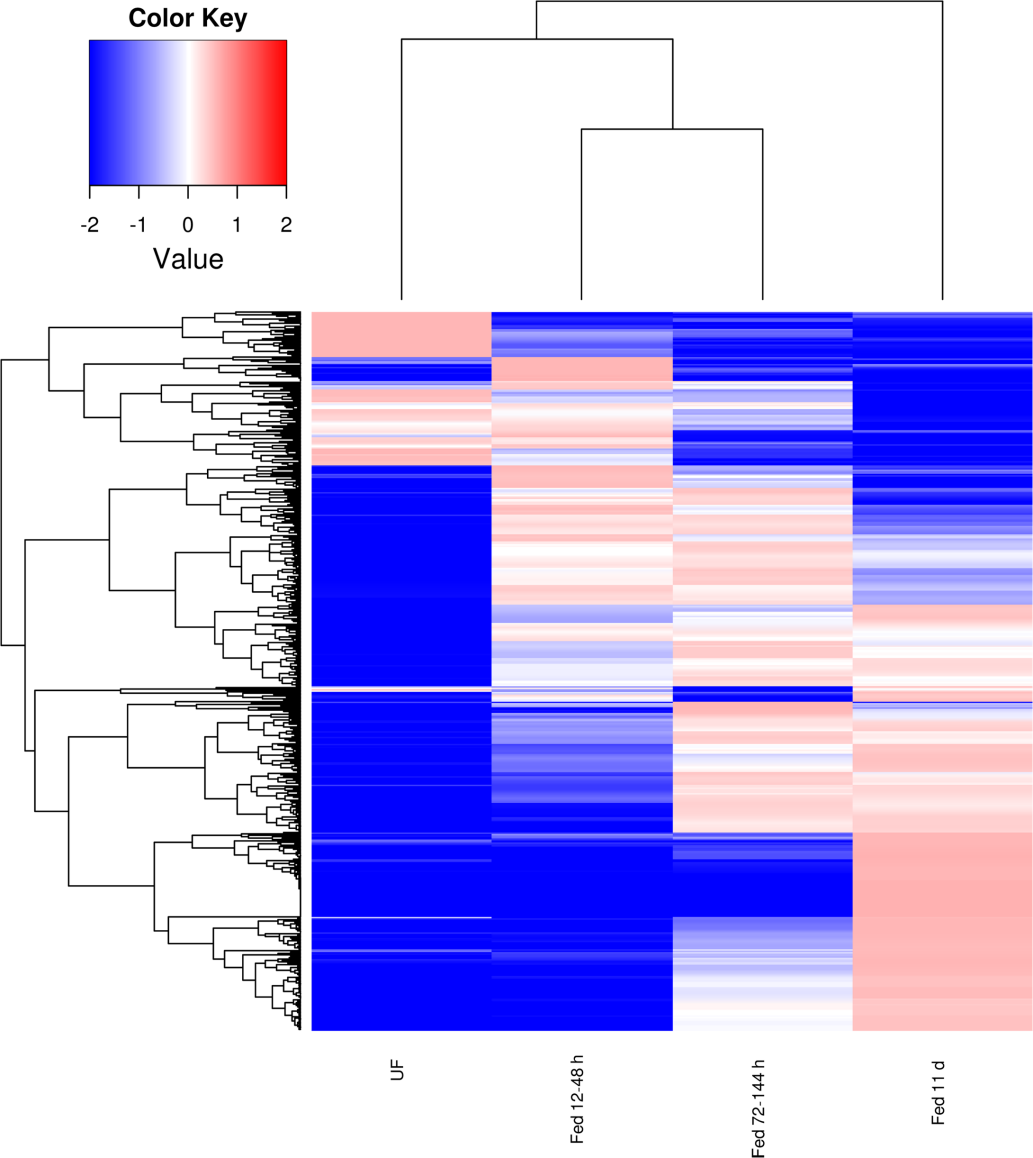
Heat map displaying 836 coding sequences (CDS) that are not uniformly expressed in the four libraries and showing a standard deviation of 1 or more regarding the log(10) transformation of the average normalized data. For more details, see text.

**Fig 2:**
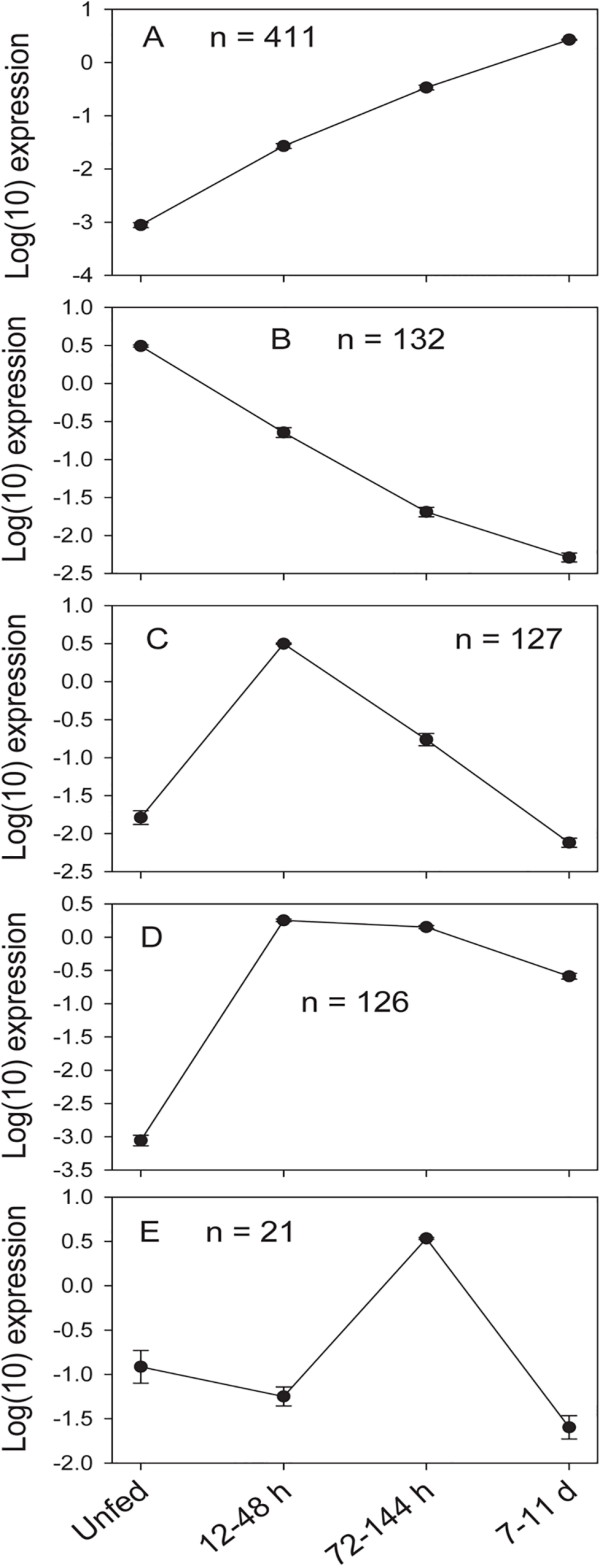
Clustering of the differential temporal expression of 817 transcripts from the *Amblyomma americanum* sialotranscriptome, using the Click algorithm of the Expander program [24]. Nineteen transcripts of the 836 shown in Fig 1 were not clustered. Each graph (A-E) represents an identified cluster. The Y axis represent the log(10) of the normalized FPKM row data, where 0 reads were substituted by 1. Symbols and bars represent the average and standard errors. For more details, see text.

Comparisons within each CDS of the number of mapped reads or FPKM values deriving from each of the four libraries (made from unfed ticks – UF, and from ticks feeding for 12–48 h, 72–144 h and 11 days) allowed for determining their temporal expression patterns which can be visualized in a heat map, and also on S1 File, worksheet named DiffExp. Fig 1 displays 836 CDS that are significantly not uniformly expressed among the 4 libraries, as assessed by a X^2^ test, having a minimum FPKM of 5 in at least one of the 4 time points and showing a standard deviation of 1 or more regarding the log(10) transformation of their average-normalized data (Fig 1). Notice that this heat map was made with log(10) transforms, not the usual log(2) transformations. Indeed, the difference from the blue to red in the Fig. represents 10,000 fold variation. This exquisitely high differential expression of transcripts according to time post-feeding in ticks has been also observed for *Ixodes ricinus* [27]. Clusterization of these 836 coding sequences with the click algorithm of the program Expander [24], using as input the FPKM data, indicates five time dependent clusters, with exclusion of 19 singleton CDS, which could not be taken into any of the 5 clusters (Fig 2). These clusters clearly define transcripts that increase their expression towards the end of the blood meal (Fig 2A), or are overexpressed in the unfed stage (Fig 2B), at 12–48 h post attachment (Fig 2C), at all stages except unfed (Fig 2D), or are overexpressed at 72–144 h post attachment, towards the middle of the blood meal (Fig 2E).

**Fig 3:**
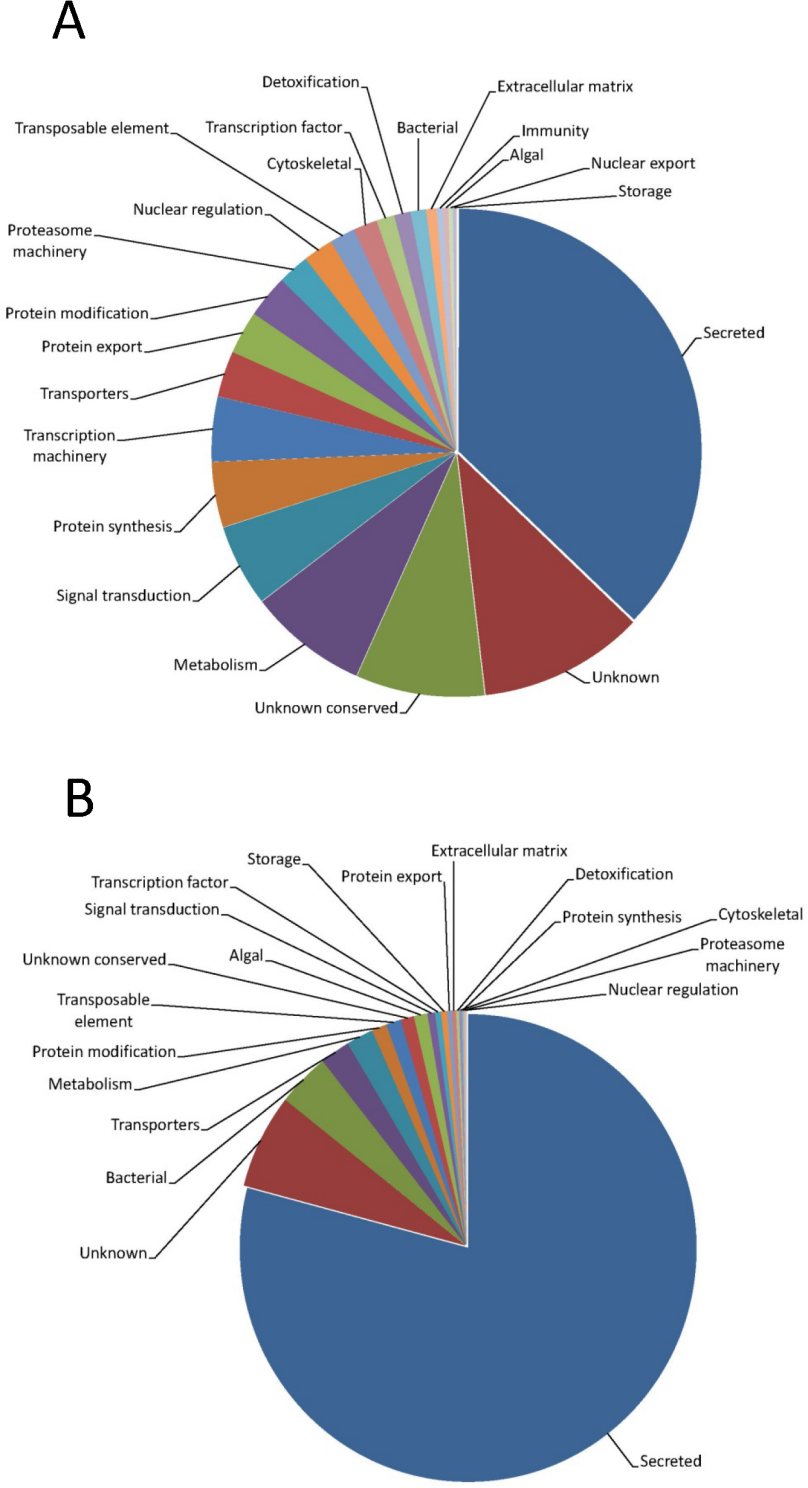
Functional classification of coding sequences and their relative abundance in the 5,792 set (A) or in the differentially expressed set of 836 sequences (B).

**Fig 4:**
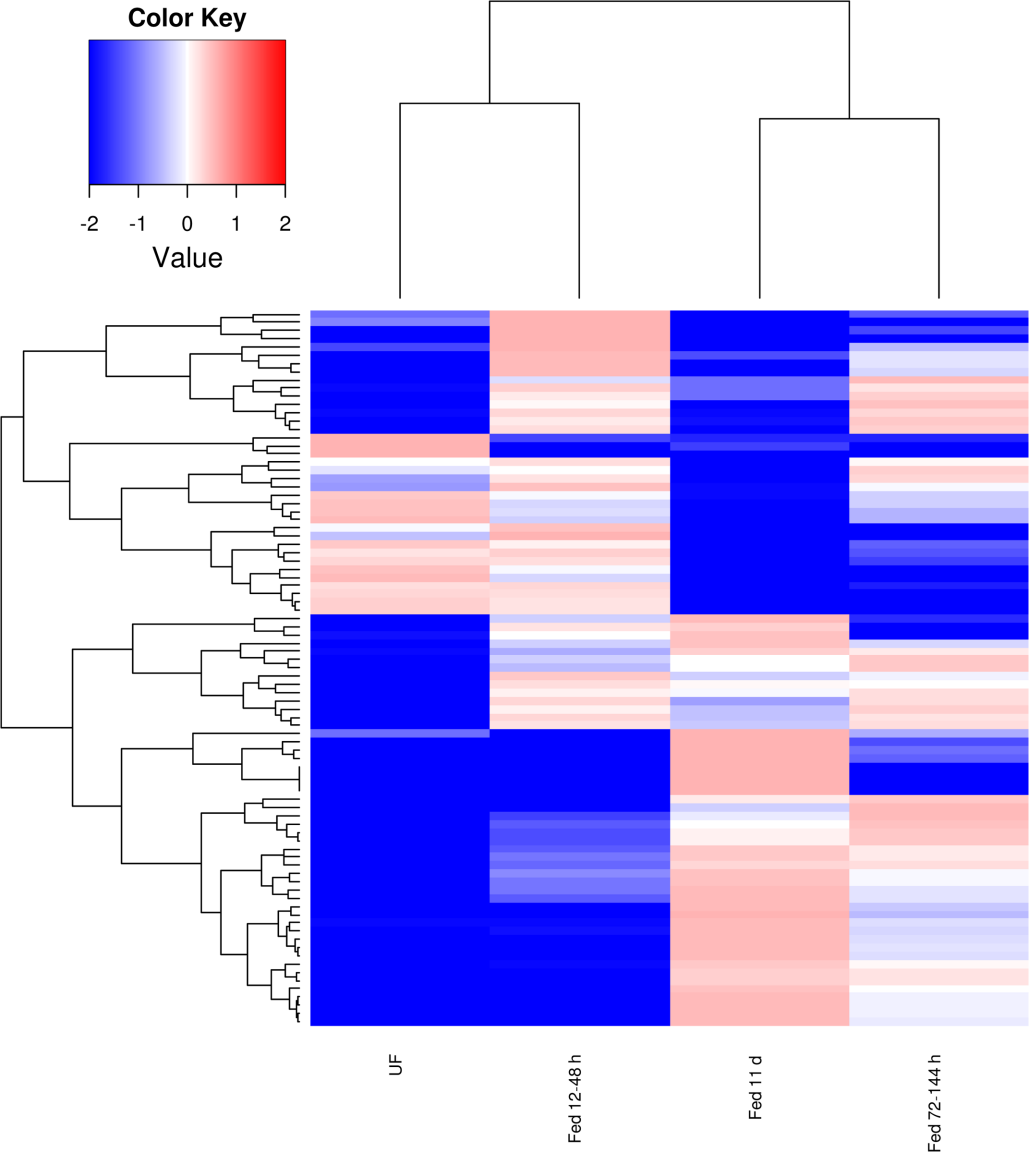
Heat map displaying 138 lipocalin coding sequences (CDS) that are not uniformly expressed in the four libraries and showing a standard deviation of 1 or more regarding the log(10) transformation of the average normalized data. For more details, see text.

**Fig 5:**
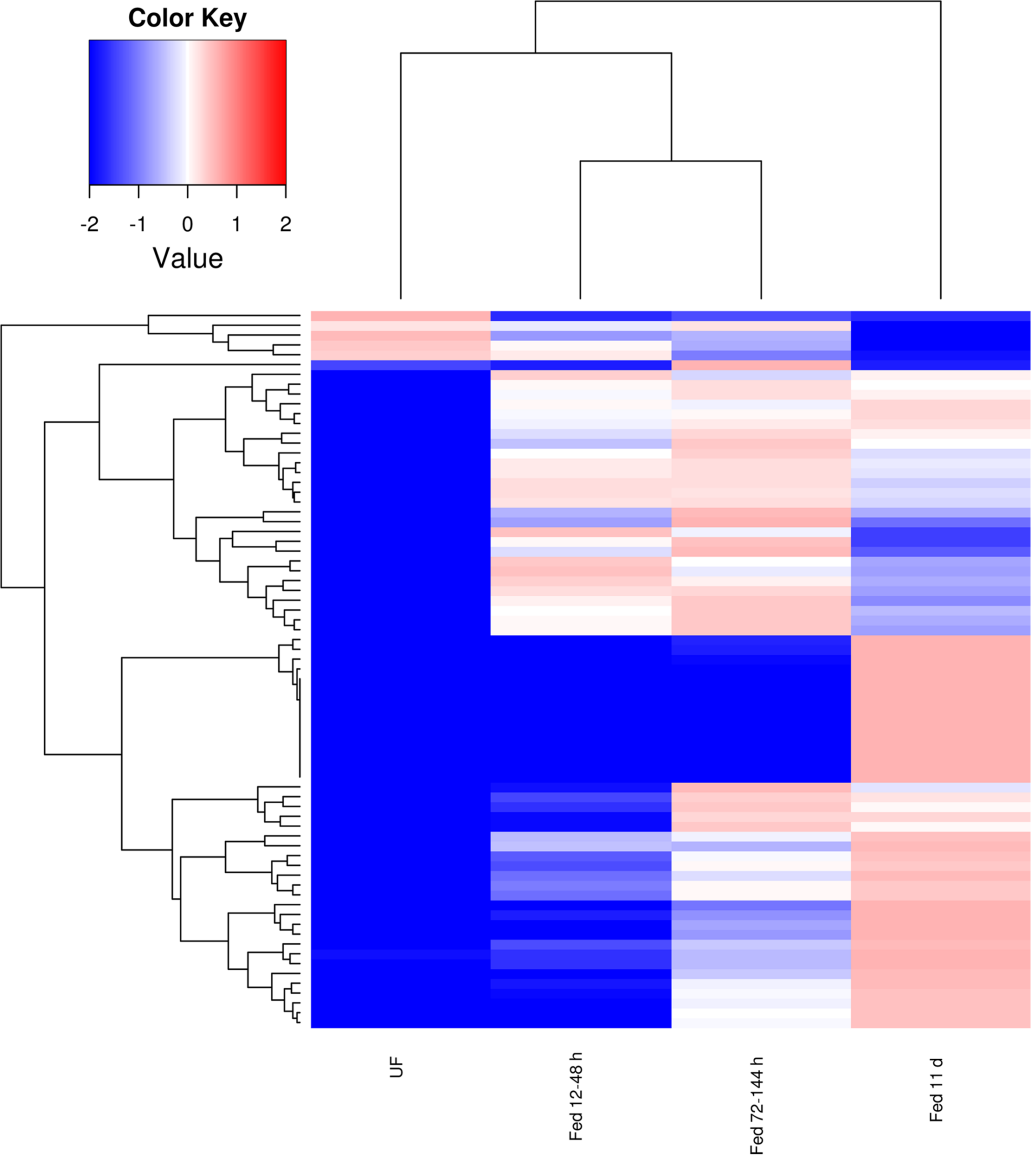
Heat map displaying 98 Kunitz domain containing coding sequences (CDS) that are not uniformly expressed in the four libraries and showing a standard deviation of 1 or more regarding the log(10) transformation of the average normalized data. For more details, see text.

While within the complete CDS set of 5,792 sequences, 37% belong to the secreted class (Table 1), 79% of the differentially expressed set belongs to this class (Table 2 and Fig 3). Of note, known multi-gene families expressed in tick sialotranscriptomes such as lipocalins (Fig 4), Kunitz (Fig 5), Cystatins, metalloproteases, TIL domain containing peptides, members of the 8.9 kDa family and evasins [see [3] for description of these families] have CDS that are remarkably differentially expressed (S1 File, worksheet “DiffExp”). Two cystatin genes from *I*. *scapularis* have been shown to change their expression reciprocally during feeding and it was postulated that these changes may reflect a form of antigenic variation of tick proteins exposed to their hosts [28]. Overall, this pattern of differential expression of secreted proteins is extreme and supports the idea that it might have evolved as a mechanism of antigenic variation of salivary proteins.

Regarding highly differentially expressed housekeeping proteins, we identify a sulfotransferase that is up regulated toward the end of the feeding period (Aam-16214), which might be associated with detoxification of dopamine agonists of salivary secretion [29] (S1 File, worksheet “DiffExp”). Several glucose dehydrogenase enzymes peak at the first 12–24 hours and might be associated with the use of host glucose as an initial energy source. A FMRFamide-related neuropeptide (Aam-32932) is maximally expressed in the unfed group, decreasing its levels more than 100 x on the subsequent time periods examined. Proteins associated with apoptotic messages, as expected, increase toward the end of the blood meal [30], as do heme lipoprotein precursors and vitellogenin, and many transporters, including one aquaporin (Aam-38627), sugar transporters and several monocarboxylate transporters. Some transposable elements also show remarkable time dependent expression levels, some being most expressed in unfed and others at the 11 day-derived libraries.

Among the highly differentially expressed CDS, 14 best matched bacterial proteins, 13 of which best matching *Coxiella*. All these bacterial transcripts were most expressed in unfed ticks. *A*. *americanum* has been found to harbor *Coxiella* sp. at high prevalence [31]. Somewhat surprising was the finding of 11 CDS best matching plant or algal sequences, all of which are most expressed in the 72–144 h library. Whether these sequences represent artifactual contamination of the sample, derive from apicomplexa parasites or some symbiotic association remains to be determined. For the record, plant-like sequences were also found in our previous *A*. *maculatum* sialotranscriptome [19], such as that recorded at http://www.ncbi.nlm.nih.gov/protein/346465067?report=genbank&log$=prottop&blast_rank=1&RID=0DKR925H013.

### Invariant transcripts

We also evaluated those CDS that did not change their expression in the various libraries. For that goal, considering only transcripts with FPKM > 10, we divided each contig FPKM value for each library by the average FPKM of all libraries and determined the standard deviation (SD) of these transformed values. Forty four transcripts had a SD < 0.05 indicating remarkable time invariance among the libraries (Table 3). None of these transcripts belong to the secreted class, and include histones, ubiquitin related proteins, transcription factors and ribosomal proteins that are normally used to normalize transcript tissue expression in qPCR experiments. Further details regarding these transcripts can be found in the worksheet named “ConstantExpression” in the S1 File. This set of results serves to validate the differentially time expressed contigs described above, and for selection of PCR primers for control transcripts when comparing transcript expression using qPCR.

**Table 3:** Time-invariant expressed transcripts.

CDS Name	Comments	Transformed Unfed FPKM	Transformed 12–48 h FPKM	Transformed 72–144 h FPKM	Transformed 11 d FPKM	SD
Aam-33201	Eukaryotic translation initiation factor 3	1.024	0.985	0.997	0.994	0.016
Aam-1649	Uncharacterized conserved protein	0.985	0.979	1.010	1.026	0.022
Aam-39788	FKBP-type peptidyl-prolyl cis-trans isomerase	1.056	0.979	0.977	0.988	0.038
Aam-40644	ubiquinol-cytochrome C reductase complex	0.973	1.026	0.955	1.045	0.043
Aam-33476	Golgi antiapoptotic protein	1.073	0.952	0.995	0.980	0.051
Aam-42030	Alcohol dehydrogenase class III	0.978	0.943	1.063	1.016	0.052
Aam-1685	Unknown product	1.037	1.046	0.995	0.922	0.056
Aam-4480	20-hydroxysteroid dehydrogenase	1.028	1.072	0.973	0.928	0.063
Aam-11842	XTP3-transactivated protein A protein	0.941	0.962	1.085	1.012	0.064
Aam-33491	glutamate transporter EAAC1-interacting protein	1.080	0.918	0.997	1.006	0.066
Aam-40639	Seryl-tRNA synthetase	1.031	1.024	0.895	1.049	0.071
Aam-10857	Alpha-tubulin suppressor	1.094	0.923	0.997	0.985	0.071
Aam-34154	6-pyruvoyl tetrahydrobiopterin synthase	0.927	0.960	1.087	1.026	0.071
Aam-13359	Phenylalanyl-tRNA synthetase beta subunit	0.954	1.028	0.930	1.088	0.072
Aam-20641	histone H2B	1.054	0.940	0.931	1.075	0.075
Aam-40212	transcription factor IIH (TFIIH)	1.074	0.991	1.039	0.896	0.077
Aam-35707	Translation machinery-associated protein	0.951	0.950	1.113	0.986	0.077
Aam-27753	Unknown product	1.091	0.928	0.937	1.045	0.081
Aam-34324	Histone H4	1.104	0.903	0.984	1.010	0.083
Aam-8796	Predicted thioesterase	0.942	1.072	1.074	0.912	0.085
Aam-39279	Unknown product	0.997	0.972	1.118	0.913	0.086
Aam-40478	28S ribosomal protein S24	0.904	1.036	0.958	1.102	0.087
Aam-36097	39S ribosomal protein L50 mitochondrial	0.945	0.986	0.940	1.129	0.088
Aam-18940	Glyoxylate/hydroxypyruvate reductase	0.999	0.883	1.018	1.100	0.089
Aam-29306	Unknown product—Membrane anchor detected	0.977	0.978	1.127	0.917	0.090
Aam-29839	ubiquitin	1.019	1.119	0.933	0.929	0.090
Aam-27902	transcription factor MBF1	1.100	0.882	1.010	1.008	0.090
Aam-22	Yippee zinc-binding protein	0.947	0.922	1.135	0.996	0.095
Aam-3708	ribonuclease P protein subunit p40-like	1.103	0.873	1.015	1.009	0.095
Aam-20820	E3 ubiquitin-protein ligase DTX4-like	0.891	1.031	0.962	1.116	0.096
Aam-1715	E3 ubiquitin-protein ligase RNF180-like	1.129	0.934	1.019	0.918	0.097
Aam-8383	transcription initiation factor TFIID subunit 8	1.145	0.948	0.947	0.960	0.097
Aam-8902	splicing factor SC35	0.872	1.107	0.996	1.025	0.097
Aam-35183	Conserved plasma membrane protein	1.044	1.116	0.904	0.937	0.098
Aam-35355	signal peptidase 12kDa subunit	1.144	0.919	0.985	0.952	0.100

FPKM were average-transformed (divided by the row average).

## Conclusions and Perspectives

This work provides for an increase from 142 to 3,281 proteins publicly deposited from *A*. *americanum* annotated as salivary proteins, thus vastly increasing the repertoire of this tick sialome that might be useful as a platform for protein discovery using mass spectrometry protocols, and to help genome annotations (gene exon/intron borders) when this tick genome is sequenced and assembled.

As found before in other time-dependent tick sialotranscriptome studies, the tick salivary secretory repertoire changes dramatically as blood feeding progresses [27, 32]. It appears that ticks have several “built-in” sialomes that are selected for expression as time progresses. Indeed, many transcripts have oscillations on the order of hundreds or thousands fold. This has been proposed before [27] as a mechanism of antigenic variation, as for example, an antigen secreted on Monday would be absent Friday and substituted by a functionally similar protein with different antigenic properties. The question remains whether the sialome switch occurs purely following an internal clock, or whether it responds to a feeding stress sensor. We have previously suggested that sialome switch in *A*. *maculatum* might follow epigenetic determined variations in histone acetylation, as serendipitously observed following the knock down of the selenocysteine elongation factor that led to an enormous increase of a sirtuin transcript involved in histone acetylation, and a dramatic change in transcripts associated with secreted salivary products [33]. This hypothesis might be tested by determining the sialotranscriptome of ticks fed artificially where no feeding stressors should change with the meal, compared to transcriptomes of “in vivo” feeding controls. Gene expression patterns displayed by ticks in experiments with naïve and successively infested hosts could also help to elucidate this matter. These sialome variations thus bring challenges to applied research related to tick vaccine antigen selection, as well as to basic research related to the underlining mechanism of sialome switching in ticks.

## Supporting Information

S1 File Hyperlinked Excel spreadsheet containing the analyzed coding sequences. (XLSX)
